# Cutting-Edge Biomaterials in Intervertebral Disc Degeneration Tissue Engineering

**DOI:** 10.3390/pharmaceutics16080979

**Published:** 2024-07-24

**Authors:** Yifan Wang, Chuyue Zhang, Junyao Cheng, Taoxu Yan, Qing He, Da Huang, Jianheng Liu, Zheng Wang

**Affiliations:** 1 Department of Orthopedics, Chinese PLA General Hospital, Beijing 100853, China; wyfspine@163.com (Y.W.); cycycyzhang301@163.com (C.Z.); cjyspine@163.com (J.C.); yantaoxu0409@163.com (T.Y.); 2 College of Biological Science and Engineering, Fuzhou University, Fuzhou 350108, China; 230820068@fzu.edu.cn (Q.H.); huangda@fzu.edu.cn (D.H.)

**Keywords:** biomaterials, intervertebral disc, intervertebral disc degeneration, tissue engineering

## Abstract

Intervertebral disc degeneration (IVDD) stands as the foremost contributor to low back pain (LBP), imposing a substantial weight on the world economy. Traditional treatment modalities encompass both conservative approaches and surgical interventions; however, the former falls short in halting IVDD progression, while the latter carries inherent risks. Hence, the quest for an efficacious method to reverse IVDD onset is paramount. Biomaterial delivery systems, exemplified by hydrogels, microspheres, and microneedles, renowned for their exceptional biocompatibility, biodegradability, biological efficacy, and mechanical attributes, have found widespread application in bone, cartilage, and various tissue engineering endeavors. Consequently, IVD tissue engineering has emerged as a burgeoning field of interest. This paper succinctly introduces the intervertebral disc (IVD) structure and the pathophysiology of IVDD, meticulously classifies biomaterials for IVD repair, and reviews recent advances in the field. Particularly, the strengths and weaknesses of biomaterials in IVD tissue engineering are emphasized, and potential avenues for future research are suggested.

## 1. Introduction

In contemporary society, the prevalence of low back pain (LBP) and its consequent disability rates are steadily escalating, imposing a substantial economic burden worldwide [1,2]. The etiology of LBP is multifaceted, with histological and imaging evidence indicating a correlation with intervertebral disc degeneration (IVDD). Common treatments include conservative approaches and surgical interventions [3]. Conservative approaches involve rest, non-steroidal anti-inflammatory drugs (NSAIDs), and physical rehabilitation, offering partial relief from pain symptoms but failing to halt IVDD progression. Moreover, their efficacy and duration are limited, with some cases showing no symptom alleviation at all [4,5]. When conservative measures prove ineffective, surgical intervention becomes the primary recourse. Surgical options such as discectomy, decompression, and spinal fusion yield significant symptom improvement. Despite the prevalence of minimally invasive spinal surgery, stringent indications must be adhered to. Furthermore, regardless of the procedure chosen, there remains a risk of increased trauma, recurrence, reduced mobility, and adjacent vertebral diseases [6,7].

Given the constraints of conservative and surgical treatments and the deepening comprehension of IVDD pathophysiology, biomaterial-based biological therapy offers novel avenues for IVDD treatment. Biomaterials such as hydrogels, microspheres, microneedles, and microfluidic chips boast remarkable biocompatibility, biodegradability, and swelling rates. Additionally, they can facilitate the delivery of cells, cytokines, drugs, genes, etc., and have found widespread application in bone, cartilage, skin, and other tissue repairs, with a growing focus on disc repair [8,9,10].

This review provides a concise introduction to the intervertebral disc (IVD) structure and the mechanisms underlying nucleus pulposus (NP) degeneration. It meticulously categorizes biomaterials for IVD repair and examines recent advances in the field.

## 2. Structure of IVD and Pathophysiology of IVDD

IVD is located between adjacent vertebral bodies and consists of three main components: a central NP, an outer annulus fibrosus (AF), and a cartilage endplate (CEP) [11,12]. The nucleus pulposus, situated between adjacent CEPs and forming the gelatinous core of IVD, is primarily composed of type II collagen and proteoglycans. The hydrophilicity of its internal structure ensures the water content and osmotic pressure in a healthy IVD, maintaining the disc’s shape and hydrostatic pressure [13,14]. The AF consists of numerous highly oriented collagen fibers that form a lamellar structure in alternating layers. Abundant in type I collagen, the AF is segmented into inner and outer layers to endure the high stress exerted by the central NP and the flexion and lateral bending of the spine [15,16]. The CEP, composed of hyaline cartilage, is crucial for maintaining disc integrity and separating the vascularized tissue from the non-vascularized tissue within the IVD. Additionally, the CEP serves as the main route for nutrients to enter the NP from the vertebral body [17]. The primary functions of the IVD are to support body weight, resist pressure, facilitate vertebral motion, and connect adjacent vertebral bodies. Both the NP and inner AF tissues have minimal blood supply, relying mainly on the diffusion of oxygen and nutrients through capillaries in the outer AF tissue and CEP [18,19]. Consequently, the IVD has limited capacity for self-regeneration and repair following degeneration.

The pathophysiological mechanisms of IVDD are still under debate and depend on various factors, including age, mechanical load, hormones, and genetics [19]. A key aspect of degeneration is the loss of IVD homeostasis, which is marked by a decrease in NP cells and alterations in the extracellular matrix (ECM) components. Endplate calcification obstructs the nutrient supply to NP cells, leading to an accumulation of metabolites and proinflammatory factors such as IL-1β, IL-6, and TNF-α. This process also results in increased levels of matrix metalloproteinases (MMPs) and reactive oxygen species (ROS), ultimately causing phenotype changes, senescence, and apoptosis of NP cells, as well as enhanced ECM catabolism and reduced ECM synthesis [20,21,22]. Additionally, the elevated expression of inflammatory factors can stimulate the formation of nerves and blood vessels, which is associated with discogenic pain. IVDD involves a wide range of cellular and molecular signaling pathways, including PI3K, NF-κB, MAPK, and ERK [23,24]. Clinically, IVDD is manifested in imaging studies by reduced MRI signals in IVD tissue, narrowing of the IVD space, irregularity and hardening of the CEP, annular herniation of the intervertebral disc, and the formation of vertebral osteophytes. These changes can lead to motor dysfunction and pain [25,26].

## 3. Mechanical Properties of IVD

AF and NP serve distinct functions in the biomechanical performance of IVD. The collagen fibers within AF not only extend into the vertebral bone but also link the adjacent vertebral bodies, thereby stabilizing the spine and establishing a connection between AF and vertebral bodies [27,28]. The dense collagen fibers in the outer lamellar regions of AF form intricate cross-folds, which effectively resist substantial tensile stresses, thereby preventing extensive disc herniation and reducing spinal strain caused by axial compression, torsion, bending movements, and axial rotation loads. Prolonged exposure to high levels of axial loading, particularly in an upright posture, leads to the compression of IVD, causing the expulsion of interstitial water. Consequently, the disc height decreases and may lead to herniation [29,30].

The nucleus pulposus, under static loads, primarily functions as a liquid, generating significant fluid hydrostatic pressure and providing a barrier to limit deformation. Due to the cartilage-like characteristics of the inner fibrous ring and NP, which are characterized by higher protein polysaccharide content as well as high water content, the entire IVD exhibits viscoelastic behavior. In the inner fibrous ring and NP, a less dense collagen matrix allows for a significant degree of deformation to accommodate loads, resulting in volume changes and subsequent flow of interstitial fluid within the disc, dissipating energy and viscoelastic creep. Degeneration of NP leads to reduced disc height and stiffness, along with increased deformation of AF. Injection of physiological saline into NP can temporarily increase intradiscal pressure and reduce segmental mobility. This demonstrates that removing NP or damaging AF alone results in loss of hydraulic function, leading to the load-deformation response of the entire IVD [31,32].

Therefore, IVD provides both flexibility and stability to the spine while also possessing biomechanical capabilities to absorb and disperse high loads. Any abnormalities in any part of IVD will affect its overall biomechanical performance. An adequately designed carrier with suitable mechanical properties is indispensable in IVD tissue engineering [33,34].

## 4. Biomaterials in IVD Tissue Engineering

**Figure 1:**
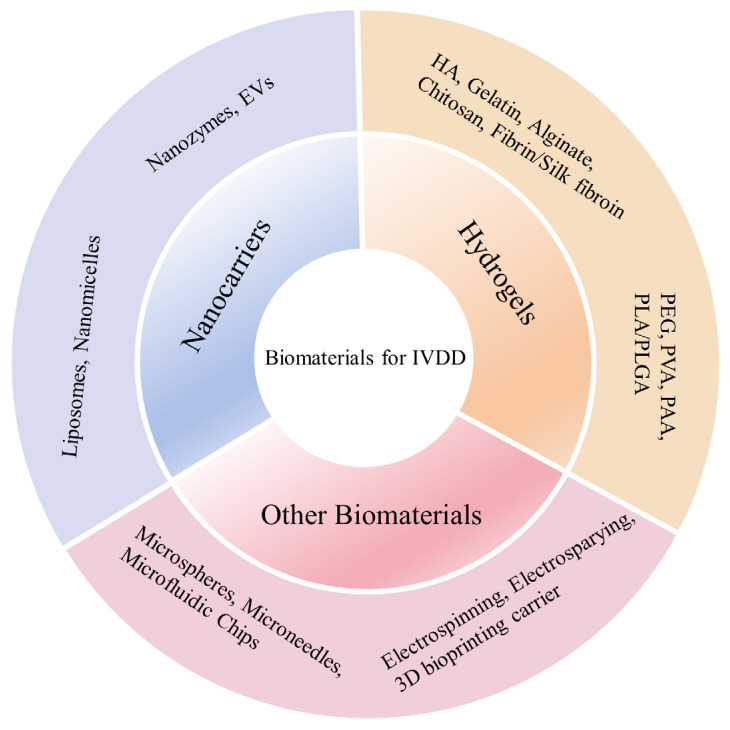
Biomaterials used in intervertebral disc degeneration tissue engineering. Abbreviations: HA, hyaluronic acid; PEG, polyethylene glycol; PVA, polyvinyl alcohol; PAA, polyacrylic acid; PLA, poly(lactic acid); PLGA, poly(lactic-coglycolic acid); EVs, extracellular vesicles; IVDD, intervertebral disc degeneration.

With the growing understanding of the anatomical structure, pathophysiology, and mechanical properties of IVD, biomaterial-based repair strategies for IVDD have garnered increasing attention. Firstly, biomaterials used for IVDD repair must exhibit excellent biocompatibility and biodegradability. This ensures they are non-toxic to host tissues and foreign cells and can match the metabolic rate and tissue regeneration processes [35,36]. Secondly, these materials must possess suitable mechanical properties to restore vertebral space height, withstand mechanical loads, and maintain spinal motion, thereby ensuring adequate load distribution. Another crucial but often overlooked parameter is the swelling rate, which reflects the hydrophilicity of the gel [37]. High-swelling materials facilitate the diffusion and release of drugs or cytokines but tend to have weaker mechanical strength. Reducing the swelling rate can stabilize the mechanical properties, and the release capabilities can be adjusted through material modifications [38,39]. In addition, the microenvironment of IVDD is complex, characterized by increased reactive oxygen species (ROS) and decreased pH. Therefore, biomaterials should not exacerbate these conditions but instead should be engineered to perform specific functions in response to these environmental factors [10]. Biomaterials such as hydrogels, microspheres, and microneedles are particularly suitable due to their modifiable properties and mechanical characteristics similar to those of biological tissues. Many of these materials are injectable and have crosslinking capabilities, making them easy to design and use [39,40]. Moreover, advanced biomaterials can encapsulate cells, deliver drugs and cytokines, and provide a controlled release to influence molecular signaling pathways, exerting anti-inflammatory, antioxidant, or other therapeutic effects. These properties make them highly effective for the regeneration and repair of IVDs [10,37]. The various types of biomaterials used for IVDD are described below (Figure 1).

## 5. Hydrogels

Hydrogels, composed of interconnected natural or synthetic polymers holding water within their structures, typically surpass particles in size [41,42]. These versatile materials operate as carriers for cells, drugs, genes, and cytokines. Significantly, hydrogels comprise fine particles, offering adaptable physical and chemical characteristics by modifying composition and concentration. This flexibility facilitates efficient drug delivery while maintaining mechanical properties that closely resemble those of the Intervertebral Disc (IVD), consequently aiding in the preservation of the IVD’s structural integrity [43]. By employing different gelation strategies, hydrogels can intelligently respond to various microenvironments, enabling accurate drug release. This capability has already been demonstrated in numerous hydrogel materials used for wound hemostasis and bone defect repairs [44]. Various materials are utilized to construct hydrogels, including natural derivatives like hyaluronic acid, alginate, and chitosan, as well as synthetic materials such as polyethylene glycol (PEG), polyvinyl alcohol (PVA), and poly(lactic-coglycolic acid) (PLGA) [36].

### 5.1. Natural Hydrogels

#### 5.1.1. Hyaluronic Acid

Hyaluronic acid (HA) is a natural polymer classified within a family of diverse heteropolysaccharides identified as glycosaminoglycans (GAGs), is found in the interstitial fluid or can be obtained through microbial fermentation [45,46]. At the normal pH level, every carboxylic group within the structure of hyaluronic acid bears a negative charge. This property allows hyaluronic acid to establish hydrogen bonds with water molecules by utilizing its carboxyl and acetamido groups, thereby enhancing the stability of the biopolymer’s secondary structurer [47]. Its biocompatibility, immunocompatibility, and susceptibility to chemical modifications make HA an ideal biomaterial choice. However, insufficient mechanical properties have constrained the applicability of HA. Consequently, the method of chemical modification and crosslinking with diverse performance-enhancing materials has emerged as a broadly acknowledged strategy [48].

**Figure 2:**
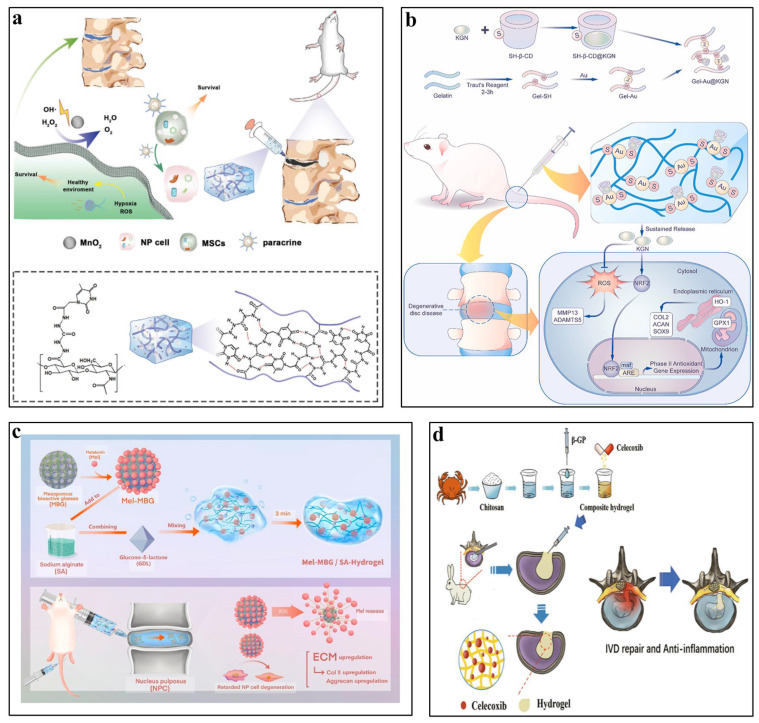
Application of natural hydrogels in IVDD tissue engineering. (**a**) Nucleobase-driven self-gelled hyaluronic acid-based injectable adhesive hydrogel [49]. Reproduced with permission from Copyright 2024 Wiley. (**b**) Dynamic self-healing gelatin hydrogel loaded with Kartogenin [58]. Reproduced with permission from Copyright 2023 Elsevier. (**c**) Injectable mesoporous bioactive glass/sodium alginate hydrogel loaded with melatonin [65]. Reproduced with permission from Copyright 2023 Elsevier. (**d**) Thermosensitive injectable chitosan hydrogel loaded with celecoxib [69]. Reproduced with permission from Copyright 2022 Frontiers.

Yang et al. [49] introduced an innovative self-gelling method driven by nucleobases in HA. By embedding nucleobases into HA chains, this approach stimulates gel formation by strengthening intermolecular hydrogen bonds. The resulting gel demonstrates self-gelation properties, injectability, and tissue adhesion capacity. Moreover, the integration of manganese dioxide (MnO2) nanoparticles aids in managing oxidative stress and addressing the hypoxic microenvironment in IVDD, thus promoting cell viability within the encapsulated environment. (Figure 2a). Chen et al. [50] developed an oxidized HA hydrogel by synthesizing aldehyde-functionalized HA (HA-CHO) and crosslinking it with gene carrier amine-terminated Generation 5 poly(amidoamine) dendrimers loaded with siRNAs. This process formed a gel through dynamic Schiff base bonds, resulting in an injectable hydrogel. This novel system significantly reduced IVD inflammation and slowed the progression of IVDD by disrupting the STING-NF-κB pathway, demonstrating the considerable potential for gene delivery applications in various diseases.

#### 5.1.2. Collagen and Gelatin

Collagen, a vital constituent of the extracellular matrix (ECM) and a naturally occurring biomaterial found abundantly in bone and soft tissues, exhibits low immunogenicity. Additionally, collagen possesses favorable biocompatibility, biodegradability characteristics, and weak cytotoxicity [51]. Gelatin, which is obtained through the heat-induced denaturation of animal collagen, retains the key sequence characteristic of collagen. This sequence is instrumental in promoting cell cycle progression [52]. Degradation products of gelatin, originating from the same collagen, also demonstrate non-toxic and non-immunogenic properties [53]. Gelatin hydrogels are often physically crosslinked, making them unsuitable for long-term applications. A representative reaction product is methylacrylylated gelatin, which is modified by methacrylate to form covalently crosslinked hydrogels. The storage modulus can be adjusted between 1.2 and 15.9 kPa by changing the degree of methacrylate substitution and the concentration of methylacrylyl gelatin, which greatly improves its mechanical strength [54]. As a result, hydrogels made from collagen and gelatin have been extensively utilized in the field of tissue engineering due to their diverse applications [55]. Hydrogel systems based on gelatin and collagen have made significant advancements in tissue engineering applications for bone, cartilage, skin, and numerous other tissues [56,57]. 

Tian et al. [58] devised a dynamic self-healing gelatin hydrogel infused with kartogenin (KGN). The administration of KGN-enhanced hydrogel effectively countered IVDD and alleviated inflammation by stimulating NRF2, which plays an essential role in reducing ROS and preventing ECM degradation in a rat model of puncture-induced IVDD (Figure 2b). In a separate study, Luo et al. [59] developed an injectable bioorthogonal hydrogel (BIOGEL) based on gelatin. Encapsulation of TGF-β within BIOGEL significantly bolstered tissue repair processes, including tissue structure and matrix remodeling, while also enhancing functional recovery, such as improved water retention by promoting matrix remodeling and reduced pain, in an in vivo rat IVDD model.

#### 5.1.3. Alginate

Alginate, a linear polysaccharide derived from natural mucopolysaccharides found in brown algae, is widely recognized for its versatility in drug delivery systems. Its biocompatibility, low immunogenicity, water retention properties, and mechanical strength make it an ideal choice for supporting cell growth and proliferation [60]. Alginate scaffolds utilize their porous structure to effectively encapsulate drugs, enabling gradual release over time. The most commonly used form of alginate in hydrogels is sodium alginate (SA) [61,62]. Moreover, sodium alginate gels with sensitivity to light, heat, pH, and ionic strength can be engineered to achieve precise control over drug release, leveraging alginate’s inherent responsiveness to these environmental factors [63]. Meanwhile, the mechanical supporting effect of sodium alginate alone is limited. Crosslinking with other biomaterials can result in alginate gels having a skeletal network structure and improved mechanical properties [54].

Li et al. [64] developed an injectable SA hydrogel reinforced with silk fibroin nanofibers featuring core–shell structures. Platelet-rich plasma (PRP) was integrated into the core–shell nanofibers to facilitate sustained release and enhance NP regeneration. In rat models of IVDD, radiographic signal intensities significantly decreased following 8 weeks of injection with the SA hydrogel. Wu et al. [65] introduced a novel injectable composite hydrogel named Mel-MBG/SA, comprising SA, melatonin, and mesoporous bioactive glasses. This composite hydrogel provides a combined system for sustained melatonin release, effectively mitigating IL-1β-induced oxidative stress and alleviating inflammation associated with IVDD pathophysiology. This innovative system presents a novel approach to promote tissue regeneration by modulating the inflammatory environment while leveraging the mechanical properties of the material (Figure 2c). 

#### 5.1.4. Chitosan

Chitosan (CS), a sole naturally occurring alkaline polysaccharide that carries a positive charge, finds extensive applications in drug delivery, antibacterial tissue engineering, and wound hemostasis [66]. Structurally akin to natural glycosaminoglycans, CS and its degradation products boast non-toxic, non-immunogenic properties and excellent biocompatibility [67]. However, the elastic modulus and compressive strength of CS hydrogels fall significantly below those of the spinal structure, necessitating additional modifications to bolster their mechanical robustness. This often entails the formation of double-network hydrogels to fulfill the intended functions [68].

Du et al. [69] synthesized a thermosensitive injectable chitosan hydrogel loaded with celecoxib. The hydrogel demonstrated low toxicity, biodegradability, and excellent biocompatibility. In animal experiments, this composite hydrogel effectively filled local tissue defects, maintaining spinal stability and slowing the progression of IVDD post-surgery (Figure 2d). Moreover, as hydrogels based on CS have gained recognition as effective carriers for exosomes due to their notable biocompatibility, biodegradability, and antibacterial capacity, Guan et al. [70] developed a hydrogel using quaternized chitosan (QCS) and oxidized starch (OST) embedded with Exos for IVDD treatment. In a rat model of IVDD, the QCS-OST/Exos hydrogel rejuvenated NP cell degeneration, facilitated ECM synthesis, and partially restored the structures of NP and AF.

#### 5.1.5. Fibrin and Silk Fibroin

Fibrin, composed of fibrinogen and thrombin, serves as a hemostatic agent widely utilized in surgery due to its role in the physiological blood coagulation cascade [71]. It is highly valued as an ideal cell carrier in bone tissue engineering owing to its exceptional biocompatibility, capacity to mitigate immunogenic responses, and abundance of integrin binding sites that facilitate cell adhesion. However, fibrin gels exhibit limited mechanical strength and often necessitate combination with other components to form a composite capable of maintaining specific mechanical properties [72].

Panebianco et al. [73] developed a genipin-crosslinked fibrin (FibGen) composite integrated with microspheres, which effectively preserved stable mechanical properties. This composite has shown promise in IVD repair by mechanisms such as apoptosis reversal and promotion of ECM synthesis.

Silk fibroin (SF), which constitutes approximately 70% of silk and is composed of 18 amino acids, flaunts remarkable mechanical characteristics that outperform those of other natural materials, with a tensile strength several times greater than Kevlar—a benchmark in the realm of high-performance fiber technology [74,75,76]. SF hydrogels exhibit significantly improved mechanical properties, which is related to the β-folding structure in the crystallization area of the protein. Due to its inherent mechanical stability, biocompatibility, biodegradability, and bioresorbability, SF has been widely utilized in tissue engineering [77].

Bhunia et al. [78] developed self-assembled SF hydrogels using natural SF derived from Bombyx mori (BM) and Antheraea assamensis (AA) without the need for external intervene. They observed that NP cells cultured on the surface of the SF hydrogels proliferated normally, and NP cells encapsulated within the SF hydrogel survived for more than seven days. These findings confirm that SF hydrogel possesses excellent cytocompatibility, making it a promising candidate for IVDD therapy.

### 5.2. Synthetic Hydrogels

#### 5.2.1. PEG 

Polyethylene glycol (PEG), a versatile synthetic polymer synthesized from polyethylene oxide and water, enjoys widespread application due to its exceptional biocompatibility, remarkable ability to retain water and diverse functionalities. Furthermore, it is notably non-allergic, meaning it poses a minimal risk of triggering allergic responses in clinical settings [79]. Leveraging these properties, some studies have utilized PEG hydrogels to develop hemostatic materials with high biocompatibility and excellent swelling rates, making them effective for rapid hemostasis [80,81,82]. PEG itself has strong mechanical properties, and its end groups can be modified by different reactions. Through these chemical crosslinking reactions, relatively strong hydrogel network structures can be obtained. PEG hydrogels also demonstrate excellent biodegradability, breaking down into harmless metabolites, which reduces the implant burden on the body [83]. The degradative characteristics of PEG-based hydrogels can be meticulously regulated by fine-tuning parameters like the length of the PEG chains, the concentration of crosslinking, and the attributes of the copolymers involved. However, due to their biodegradability, PEG hydrogels, while exhibiting some inherent instability, can have their durability significantly enhanced through the strategic incorporation of supplementary crosslinking or innovative modifications, thereby extending their functional longevity [84].

**Figure 3:**
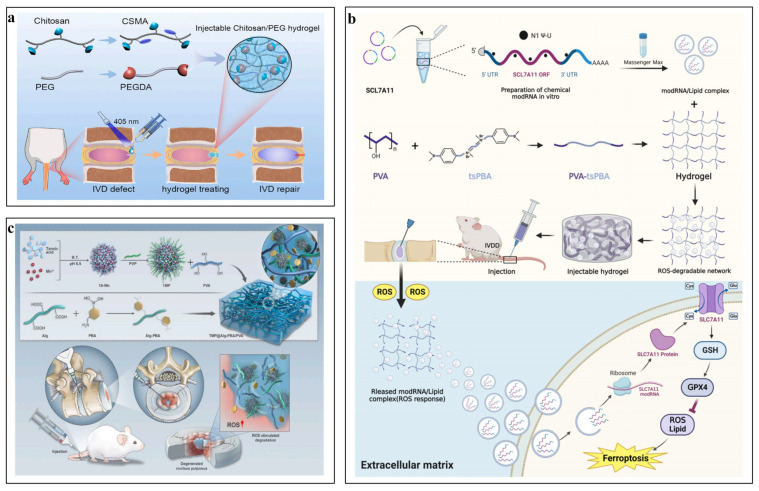
Application of synthetic hydrogels in IVDD tissue engineering. (**a**) Rapidly in situ forming an injectable chitosan/PEG hydrogel [85]. Reproduced with permission from Copyright 2023 Elsevier. (**b**) ROS-responsive PVA-tsPBA injectable hydrogel loaded with SLC7A11-modRNA [90]. Reproduced with permission from Copyright 2024 Wiley. (**c**) A novel biomimetic proteoglycan (BPG) by covalently linking natural chondroitin sulfate (CS) brushes to a synthetic PAA core [97]. Reproduced with permission from Copyright 2018 Elsevier.

Huang et al. [85] introduced an innovative, rapidly forming injectable hydrogel (CSMA-PEGDA-L) composed of chitosan and PEG, which is created through the photo-crosslinking of methacrylate chitosan (CSMA) and a Schiff base reaction between CSMA and aldehyde polyethylene glycol (PEGDA), achieved by substituting 4-formylbenzoic acid for the hydroxyl group on the PEG chain. When applied to punctured IVDs in rat tails, the CSMA-PEGDA-L hydrogel significantly decelerated the development of IVDD by physically sealing the puncture sites, as evidenced by radiological and histological assessments (Figure 3a). Hossein et al. [86] developed a PEG hydrogel based on 4-arm poly(ethylene glycol)-b-poly(trimethylene carbonate)-acrylate (4a[PEG-b-PTMC-Ac]), which was crosslinked with thiolated chondroitin sulfate through a Michael-type reaction. These hydrogels have exhibited impressive resilience to cyclic compression stress and have shown a controlled degradation profile over an in vitro period of 70 days. The formulation of this in situ gelling hydrogel is highly promising, and with continued optimization, it may emerge as a potent clinical intervention for the treatment of IVDD.

#### 5.2.2. PVA 

Polyvinyl alcohol (PVA), a pristine, stable, and non-toxic polymer that is readily soluble in water, has a broad spectrum of applications spanning the realms of medical care, culinary uses, and the chemistry of polymer materials. It is frequently employed in the creation of wound dressings, materials with antibacterial properties, and as a substitute material for implants, underscoring its versatility and utility in various fields [87,88]. The formulation and preparation of PVA hydrogels can be modified to create microporous structures with exceptional elasticity and compressibility. Moreover, PVA’s ability to bind nucleic acids through its positively charged hydrogen atoms makes it an excellent candidate for encapsulating mRNA [89].

Gao et al. [90] developed an injectable ROS-responsive hydrogel designed for the swift encapsulation and targeted discharge of chemically crafted modified mRNA. This innovative PVA-tsPBA hydrogel system encapsulates modRNA to inhibit ferroptosis in NP cells, presenting a promising new strategy for patients with early-stage IVDD (Figure 3b). Zhou et al. [91] crafted an innovative, multifunctional metal–phenolic network delivery system named TMP@Alg-PBA/PVA, specifically tailored for the treatment of IL-1β-induced IVDD. This advanced platform is designed to target mitochondria, effectively neutralizing ROS and mitigating the degradation of ECM. Additionally, it curbs pyroptosis by dampening the IL-17/ERK signaling cascade. In a rat model simulating IVDD, TMP@Alg-PBA/PVA has showcased remarkable therapeutic efficacy, significantly curtailing the progression of the disease.

#### 5.2.3. PAA

Polyacrylic acid (PAA) represents a category of anionic hydrogels endowed with carboxyl groups. The intrinsic characteristics of these hydrogels, including their mechanical strength, water absorption capacity, and porosity, can be meticulously tailored by modulating the crosslinking density [92,93]. Due to the large number of carboxyl groups in the main chain, acrylic monomers can form hydrogels through covalent crosslinking and physical crosslinking, which results in a stable structure [94]. As a prevalent pH-responsive polymer, PAA frequently assumes the role of the hydrophilic component within amphiphilic or amphipathic block copolymers, bestowing these materials with distinctive attributes [95]. Owing to its minimal toxicity and high biodegradability, PAA has garnered extensive application in the realms of antimicrobial agents, tissue engineering, and cancer therapeutics [96].

Prudni kova K et al. [97] engineered an innovative biomimetic proteoglycan (BPG) by covalently attaching natural CS brushes onto a synthetic PAA core. This process has resulted in a three-dimensional structure that resembles the bottle-brush morphology of natural proteoglycans. Upon injection into bovine NP tissue, the BPG is effectively dispersed throughout the ECM and seamlessly integrates at the molecular level. This novel biomaterial has demonstrated remarkable resilience, being capable of withstanding both tensile and compressive forces (Figure 3c).

#### 5.2.4. PLA/PLGA

Poly(lactic acid) (PLA) and poly(lactic-coglycolic acid) (PLGA), which have non-toxic, biodegradable properties, have been approved by the U.S. Food and Drug Administration (FDA) for use in the pharmaceutical field, are classes of polymer carrier materials with great potential [98,99,100]. PLA and PLGA have become staples in targeted drug delivery systems, leveraging their distinctive biodegradability, affinity for adsorption, and exceptional biocompatibility. Compared with PLA, PLGA has obvious advantages in uniaxial tensile properties, compressive properties, and three-point bending properties [101,102].

Hydrogels containing PLA/PLGA are now widely applied in tissue engineering, with PLGA-PEG-PLGA being particularly noteworthy. This block polyester polymer is synthesized through ring-opening polymerization (ROP) using PLGA and PEG as initiators [103,104,105]. Chen et al. [106] developed an injectable, heat-sensitive PLGA-PEG-PLGA hydrogel loaded with bevacizumab, a vascular endothelial growth factor (VEGF) inhibitor. Their study demonstrated that inhibiting VEGF expression with bevacizumab decreased MMP3 expression and promoted COL II synthesis, thus improving disc degeneration. In addition, Marshall et al. [107] have ingeniously modified PLA to develop a variant known as flexible PLA (FPLA). Utilizing this material, they have crafted a viscoelastic scaffold with customizable biomechanical properties, ideal for applications involving the entire spinal motion segment. These FPLA scaffolds exhibit biocompatibility, fostering the survival of NP cells within hydrogel composites. This pioneering study reveals that FPLA scaffolds exhibit newly acquired viscoelastic mechanical characteristics that closely mimic the native IVD motion segment under both tensile and compressive loads. The results underscore the substantial promise of FPLA as a mechanically robust and biocompatible material for IVD tissue engineering.

## 6. Microspheres

Microspheres, three-dimensional spherical structures ranging from 1 to 250 μm, feature crosslinked polymer networks at the nanometer scale. This architecture grants them exceptional biocompatibility and allows for easy structural adjustments, including variations in stiffness, porosity, and composition. These properties, combined with their high encapsulation efficiency for therapeutic agents such as drugs and cells, make microspheres invaluable in various biomedical applications [108,109]. Through diverse fabrication techniques, microspheres can be customized to meet specific needs, ensuring versatility in their applications. Whether they are used for delivering biologics, aiding in tissue regeneration, or facilitating bio-lubrication, their injectability and multifunctional nature make them promising tools for addressing clinical conditions. Based on the special structural characteristics of microspheres, the particle size and crosslinking density of microspheres have significant effects on the swelling behavior and mechanical properties of physically crosslinked hydrogels [110]. Additionally, their stability and slow degradation profile enable the sustained release of active ingredients, accommodating a wide range of substances from small hydrophobic drugs to large organic molecules and even cells [111,112].

**Figure 4:**
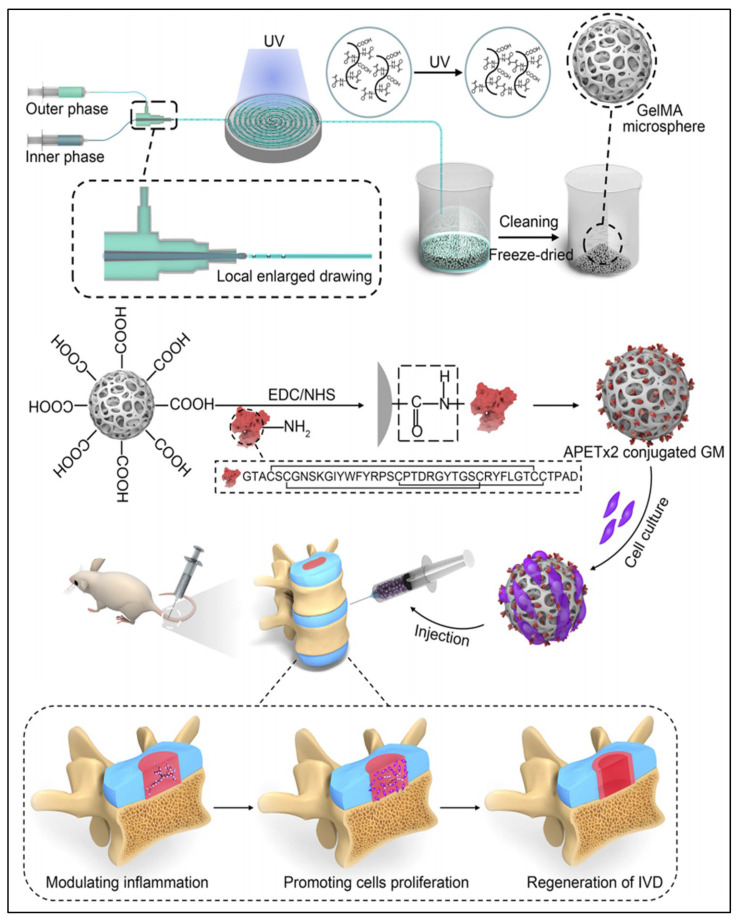
Injectable “peptide-cell-hydrogel” GelMA microsphere system [115]. Reproduced with permission from Copyright 2021 American Chemical Society.

Gelatin methacrylate anhydride (GelMA) has proven to be exceptionally biocompatible and can be meticulously crafted into uniform hydrogel microspheres through the application of microfluidic technology. The porous structure that emerges post-freeze-drying offers an enhanced surface for the integration of nanoparticles, further augmenting its utility in various applications [113]. Consequently, GelMA microspheres have gained significant attention for injectable therapies targeting IVDD. Li et al. [114] employed microfluidic technology and surface modification methods to successfully attach chitosan nanoparticles, which encapsulate potent black phosphorus quantum dots (BPQDs), onto the surface of GelMA microspheres. This groundbreaking strategy serves to alleviate extracellular acidosis in NP cells, disrupts the inflammatory response, diminishes the expression of matrix metalloproteinases (MMPs), and promotes the restructuring of ECM within IVDs. Similarly, Bian et al. [115] constructed an injectable “peptide-cell-hydrogel” microsphere system composed of GelMA, APETx2, and NP cells. This system effectively inhibits local inflammatory cytokine, thereby regulating the synthesis of ECM (Figure 4). Zhou et al. [116] successfully developed a nanoparticle–microsphere complex (GM@T-NNP cells) by integrating a biomimetic method and nano-drug delivery strategy. This complex consists of neutrophil membrane-coated PLGA nanoparticles, TGF-β1, and GelMA microspheres. GM@T-NNP cells were shown to reduce inflammation, restore IVD structure, and significantly improve the biological function of NP cells in a rat model of IVDD. In addition, Zheng et al. [117] heralded the development of a novel hydrogen ion-capturing hydrogel microsphere, termed GMNP, which incorporates mineralized TGF-β and catalase nanoparticles. This creation was achieved through a combination of biomimetic mineralization processes and the precision of microfluidic technology. The GMNP has been shown to effectively neutralize the NLRP3 inflammatory cascade, a critical pathway in the aging process characterized by chronic inflammation, thereby suppressing excessive activation of inflammation in IVD. Chen et al. [118] crafted a sophisticated system of fibronectin-modified GelMA microspheres, boasting an independently adjustable elastic modulus and ECM ligand density, offering a high degree of customization for specific applications. This design allowed for extensive cytoskeleton modulation, triggering specific signaling pathways, which presents a promising approach for the endogenous regeneration of NP cells.

In another study, Chen et al. [119] developed circadian clock-regulating microspheres (ClockMPs) made from PVA using air-microfluidic techniques and crosslinked with modified phenylboronic acid (PBA) linkers, which effectively activated the intrinsic circadian clock of NP cells in IVDD, enhancing their physiological function and promoting disc regeneration. Chang et al. [120] designed methacrylated hyaluronic acid (HAMA) microspheres using microfluidics, which exhibited excellent degradability, swellability, and injectability. HAMA microspheres loaded with CircSTC2-silenced gene-encapsulated cationic liposomes significantly promoted the synthesis of ECM-related proteins and inhibited the secretion of ECM catabolism-related proteases in NP cells. In addition, Xu et al. [121] reported the development of antagonist-functionalized injectable porous microspheres, which were constructed from bovine serum albumin nanoparticles encapsulating recombinant human soluble tumor necrosis factor receptor type II (rhsTNFRII) and microfluidic poly(L-lactic acid) (PLLA) porous microspheres for in situ injection into NP cells. This approach aimed to regulate the metabolic balance of the ECM, thereby inhibiting IVDD. 

## 7. Microneedles

Microneedles (MNs), with their micron-sized projections typically ranging from 100 to 1000 µm in length, provide a pathway for localized and minimally invasive drug delivery, which effectively addresses issues such as anchoring the AF, localized drug release, and preventing further exacerbation of IVDD [122,123]. MNs offer several advantages, including painlessness, increased depth of delivery, and efficient, long-lasting drug administration. The mechanical properties of MNs are affected by factors such as tip size, geometric shape, and number of tip arrays. Currently, MNs are mostly used in skin tissue engineering and rarely in IVD tissue engineering. Hydrogel-based MNs have garnered significant preference in the realms of drug delivery systems, attributed to their straightforward fabrication process and the flexibility in their structural design [124].

**Figure 5:**
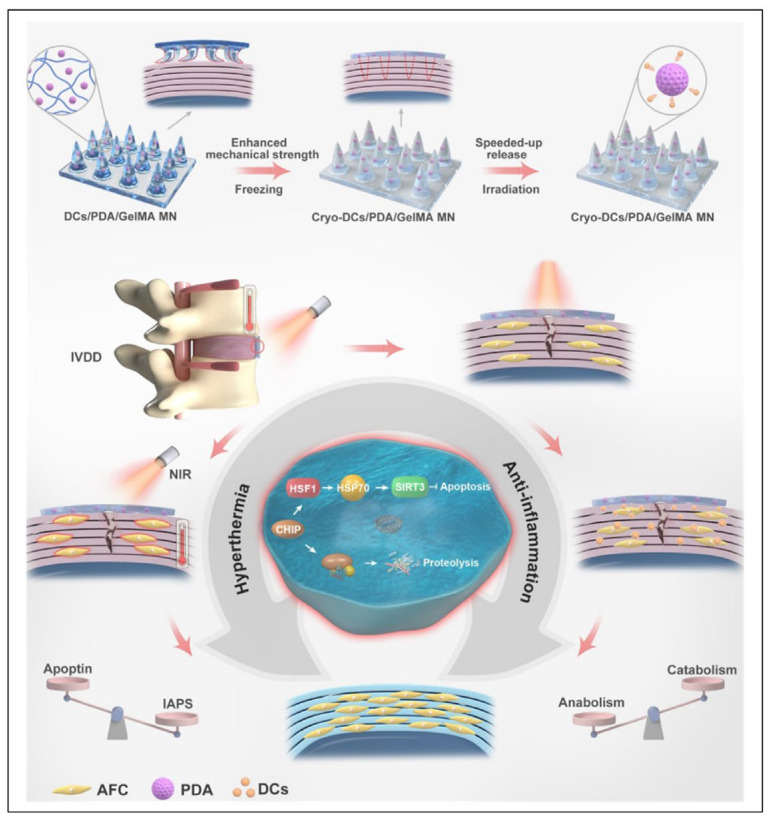
Thread-structural microneedles loaded with BMSCs-derived exosomes [125]. Reproduced with permission from Copyright 2024 Elsevier.

Meng et al. [125] developed high-strength smart polydopamine/GelMA MNs loaded with diclofenac sodium, which can penetrate AF tissue through a localized, minimally invasive method and achieve remote-controlled, accelerated drug release and hyperthermia via near-infrared light. To enable MNs to penetrate and anchor on AF, the mechanical strength of MNs was improved by cryogenic preservation methods. The composite MNs were designed to address the inflammation, mitigating cellular damage while elevating the intracellular concentration of cytoprotective heat shock proteins to bolster the cellular defense mechanisms, thereby enhancing resilience against the adverse conditions of the microenvironment (Figure 5). Hu et al. [126] reported a thread-structured microneedle (T-MN) loaded with BMSCs-derived exosomes containing a mitophagy-regulating microRNA. This T-MN offers an in situ anchorage at the AF defect site, serving as a barrier against the leakage of NP cells and fostering the repair of AF. The T-MN firmly bonds with AF, gradually releasing therapeutic engineered exosomes. It contributes to the prevention of IVDD progression through a multifaceted approach: it restores mitophagy, stimulates the proliferation and migration of AF cells, and curbs the pathological remodeling of ECM.

## 8. Microfluidic Chips

Microfluidics, the manipulation of small fluid volumes, has evolved into a versatile technology with increasingly complex chip designs [127]. Lab-on-a-chip technology, rooted in microfluidic engineering, replicates physiological activities in vitro and holds promise for developing new platforms for various disease studies [128,129].

Kim et al. [130] developed a bespoke electrical stimulation platform that is capable of delivering low-constant-current stimulation (LCCS) signals to microfluidic chips. This innovative setup facilitates the investigation of the impact of LCCS on IL-1β-induced inflammatory responses in NP cells. The research revealed that LCCS applied at current densities exerted a beneficial modulation on the morphological phenotype and kinetic behavior of the inflamed human NP cells. These findings indicate that LCCS may hold promise as a therapeutic intervention for degenerative conditions.

## 9. Electrospinning

Electrospinning, a prevalent method for crafting tissue engineering scaffolds, is adept at generating continuous microfibers or nanofibers [131]. These fibers, endowed with a high specific surface area, can significantly enhance tissue regeneration when impregnated with therapeutic drugs or cytokines. Its porosity and pore sizes facilitate cell infiltration and growth, making it advantageous for regenerating thicker tissues. The method provides favorable conditions for cell adhesion and growth due to its suitable mechanical strength and biocompatibility. The mechanical properties of electrospinning mainly depend on the molecular chain arrangement, crystallinity, and the form of interfiber connection. Additionally, various nanofiber structures can load additives, and aligned nanofibers can be collected using rotators to orient specific biomimetic tissues [132,133].

Han et al. [134] utilized electrospinning to fabricate aligned core–shell nanofibrous scaffolds, which were enriched with TGF-β3 and ibuprofen (IBU). The research findings confirmed that the swift release of IBU ameliorated the inflammatory microenvironment, while the sustained delivery of TGF-β3 facilitated the development of new ECM. This multifunctional nanofibrous scaffold has shown considerable promise as a reparative solution for IVD repair.

## 10. Electrospraying

Electrospraying, a technique leveraging electrostatic forces to fabricate nanoparticles, overcomes the harsh conditions of traditional encapsulation techniques (such as pH and temperature extremes) by generating solid particles in a one-step process without high temperatures or toxic solvents [135,136]. Recent breakthroughs in electrospraying techniques have facilitated the creation of particles with precise size specifications and uniform size distributions, all at an accelerated pace of production [137].

Leopfe et al. [138] designed microparticles encapsulating epigallocatechin-3-gallate (EGCG) using electrospraying of glutaraldehyde-crosslinked gelatin. This electrospray-based encapsulation preserved EGCG activity and produced cytocompatible microparticles capable of controlling EGCG release. The resulting microparticles showed the potential to promote IVD health by downregulating local inflammation.

## 11. Three-Dimensional Bioprinting Carrier

Three-dimensional (3D) bioprinting is a cutting-edge technology that holds tremendous promise and offers significant advantages for IVD repair [139]. As a tailored, adaptable, and exacting methodology, 3D bioprinting is capable of generating highly precise biomimetic constructs that emulate the natural architecture and functionality of indigenous tissues and organs. The mechanical attributes of these constructs can be further enhanced by incorporating a variety of distinct material components. This technology allows for the precise placement of a combination of biomaterials, cells, drugs, or cytokines [140,141].

Sun et al. [142] designed an IVD carrier incorporating polydopamine nanoparticles loaded with connective tissue growth factor (CTGF) and TGF-β3, which were combined with BMSCs to regenerate NP and AF. In vitro experiments confirmed that the BMSCs differentiated into NP-like cells and AF-like cells. In vivo studies have illustrated that the reconstructed IVD displayed a zone-specific matrix, complete with matching histological and immunological characteristics, underscoring the promising clinical applicability of the dual-growth factor-releasing IVD scaffold, which was meticulously crafted through the medium of 3D bioprinting. In addition, Yang et al. [143] developed a bioprinting technique specifically for bacterial cellulose (BC) nanofibers, utilizing a high-throughput, finely optimized micropattern screening microchip. The innovative process has yielded a complete IVD model that includes a type II collagen-rich NP and a hierarchically organized, micro-patterned AF based on BC, closely replicating the native IVD tissue structure. Experiments in rats have confirmed the superior structural and functional integrity of the synthetic IVD, which presents a groundbreaking strategy for the fabrication of highly biomimetic artificial tissues, offering a significant advancement in the field.

## 12. Nanocarriers

Nanocarriers represent the smallest delivery systems, typically utilized for the transport of small molecule drugs and genetic therapeutics [144]. From the vantage point of surface chemistry, diminishing the dimensions of nanomaterials escalates the proportion of atoms present on the surface and the incidence of lattice imperfections. This enhancement markedly boosts their surface reactivity, thereby promoting the engagement of biophysical and biochemical interactions at the bio–nano interface [145,146]. The exploitation of this distinctive attribute paves the way for the development of functional nanomaterials, broadening their utility across a spectrum of biomedical applications. Meanwhile, nanocarriers are usually loaded into the target area by injection or by carrying other biological scaffolds. Furthermore, nanocarriers often exhibit lower biological toxicity, minimizing unnecessary tissue damage and improving the safety profile of therapeutic interventions [147].

### 12.1. Liposomes

Liposomes, the trailblazing nanoparticles that have been successfully integrated into clinical practice, are spherical nanostructures encapsulated by lipid bilayers [148]. Resembling cellular membranes, they are predominantly composed of phospholipids and cholesterol. Characterized by a hydrophilic core and multiple hydrophobic regions enclosed by lipid bilayers, liposomes possess the unique capability to encapsulate a diverse array of substances, spanning hydrophobic to hydrophilic compounds [149,150].

In a study by Banala et al. [151], siRNAs targeting Caspase3 and ADAMTS5, genes associated with the degradation of ECM, have been encapsulated within cationic liposomes. These liposomal formulations not only bolster the stability of the siRNAs but also enhance their cellular uptake when administered in vivo. The direct intradiscal injection of this siRNA–liposomal complex effectively silenced the expression of Caspase3 and ADAMTS5, resulting in diminished apoptosis and curtailment of ECM degradation. This method underscores the promise of direct siRNA delivery to IVD as a noninvasive therapeutic strategy for IVDD.

### 12.2. Nanomicelles

Nanomicelles, delivery systems formed by the spontaneous self-assembly of amphiphilic polymers in aqueous solutions, can be customized to feature a spectrum of particle sizes and functionalities [152]. The micelle’s hydrophobic core serves as a reservoir for hydrophobic chemotherapeutic drugs, enhancing their aqueous solubility. Simultaneously, the micelle’s hydrophilic shell forms a protective hydration layer that shields the encapsulated agents. This protective layer minimizes protein adsorption and slows down the clearance by the reticulo-endothelial system during circulation, effectively prolonging the drug’s therapeutic half-life. Typically, nanomicelles are sized between 10 and 100 nanometers, with tight control over size distribution. The particle size can be finely tuned by modulating the lengths of both the hydrophilic and hydrophobic components of the micelles [153,154].

**Figure 6:**
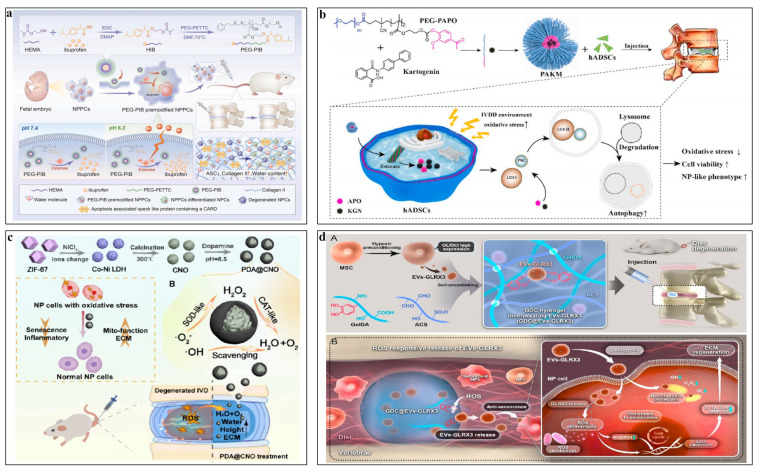
Application of nanocarriers in IVDD tissue engineering. (**a**) Biomaterial pre-modification cell strategy using esterase-responsive ibuprofen nanomicelles [155]. Reproduced with permission from Copyright 2023 Elsevier. (**b**) Injectable kartogenin and apocynin-loaded nanomicelles [156]. Reproduced with permission from Copyright 2021 Elsevier. (**c**) Nanozyme consisting of a Co-doped NiO nanoparticle (CNO) core encapsulated with a polydopamine (PDA) shell [160]. Reproduced with permission from Copyright 2024 American Chemical Society. (**d**) A redox homeostasis modulatory hydrogel with GLRX3+ extracellular vesicles [167]. (A) The GDC@EVs-GLRX3 hydrogel system was synthesized from GelDA, Borax-ACS, and EVs-GLRX3. (B) The GDC@EVs-GLRX3 hydrogel system attenuated mitochondrial damage, decreased local senescence state, and restored matrix deposition of NP cells via ROS microenvironment mitigation. Reproduced with permission from Copyright 2023 American Chemical Society.

Xia et al. [155] introduced a biomaterial pre-modification cell strategy using esterase-responsive ibuprofen nanomicelles (PEG-PIB) to pre-modify nucleus pulposus progenitor cells (NPPCs), which enhanced the adaptability of NPPCs in the harsh IVDD microenvironment by inhibiting pyroptosis. In vitro studies demonstrated that PEG-PIB pre-modified NPPCs effectively inhibited pyroptosis. Further synergistic transplantation of these modified cells resulted in functional recovery, histological regeneration, and continued inhibition of pyroptosis during IVDD (Figure 6a). Yu et al. [156] developed a novel amphiphilic copolymer, PEG-PAPO, which self-assembles into nanosized micelles capable of loading lipophilic kartogenin (KGN) as a single complex (PAKM). This innovative complex enhanced the viability of human ADSCs, activated autophagy (as indicated by P62 and LC3 II), maintained ECM-related transcription factors (such as SOX9), and preserved ECM components (including Collagen II and Aggrecan) (Figure 6b).

### 12.3. Nanozymes

Nanozymes are nanomaterials that exhibit enzyme-like properties, often mimicking superoxide dismutase, catalase, and peroxidase. Compared to natural enzymes, nanozymes provide superior physicochemical stability, enduring performance, and reduced production expenses, even under harsh environmental conditions [157]. The metal active sites within nanozymes emulate the catalytic electron transfer reactions of natural enzymes, with their catalytic potency being directly related to their size. Nanozymes that possess larger surface areas and an increased number of accessible active sites generally display enhanced catalytic activity, which allows for the precise modulation of their catalytic attributes by adjusting the dimensions of the nanomaterials [158,159].

Wang et al. [160] constructed a core–shell structured nanozyme consisting of a Co-doped NiO nanoparticle (CNO) core encapsulated with a polydopamine (PDA) shell. This nanozyme aims to regulate the pathological environment of IVD, which also effectively reduces intracellular ROS in NP cells to benign H_2_O and O_2_, thereby protecting NP cells from stagnant proliferation, abnormal metabolism, and inflammation, ultimately restoring ECM homeostasis (Figure 6c). Shi et al. [161] introduced a versatile greigite nanozyme with dual capabilities: it liberates a generous amount of polysulfides and demonstrates potent superoxide dismutase and catalase activities. These multifaceted functions are instrumental in neutralizing ROS and in sustaining tissue at its physiological redox balance. By substantially reducing ROS levels, the greigite nanozyme effectively restores impaired mitochondrial function within IVDD models in both in vitro and in vivo settings, averts the senescence of NP cells, and mitigates the inflammatory response.

### 12.4. Extracellular Vesicles

Extracellular vesicles (EVs), minuscule structures enveloped by membranes and laden with an array of biologically active molecules—including proteins, nucleic acids, and lipids—are increasingly acknowledged for their pivotal roles in a spectrum of biological phenomena. These include facilitating intercellular communication, modulating inflammation, guiding cell differentiation, and driving tissue repair [162,163]. EVs are typically categorized into exosomes and microvesicles based on their size and origin. Exosomes, with diameters spanning 30 to 150 nanometers, are generated by the body’s intrinsic multivesicular system, whereas microvesicles, measuring between 100 nanometers and 1 micrometer in diameter, emerge from the direct budding of cell membranes. Given their fundamental function in cellular dialogue and regulatory processes, extracellular vesicles (EVs) have captured considerable interest in the realms of biomedical research and clinical applications [164,165].

Liao et al. [166] developed engineered EVs by modifying caveolae-associated protein 2 (Cavin-2) through gene editing of parental mesenchymal stem cells (MSCs). These modified EVs demonstrated an improved uptake rate in TNF-α-treated NP cells, effectively preventing cell death by protecting against pyroptosis and slowing the progression of IVDD in an ex vivo organ culture model. Liu et al. [167] created GLRX3+ MSC-derived EVs (EVs-GLRX3) using a hypoxic preconditioning method, which enhanced cellular antioxidant defense, thereby preventing ROS accumulation and the expansion of the senescence cascade in vitro. EVs-GLRX3-loaded carriers mitigated mitochondrial damage, alleviated NP cell senescence, and restored ECM deposition by modulating redox homeostasis (Figure 6d). In addition, due to mechanically responsive miR-1249 is identified via miRNA sequencing to be down-regulated in compression-induced IDD of rats, Guo et al. [168] identified mechanically responsive miR-1249 as being down-regulated in compression-induced IVDD in rats through miRNA sequencing. They developed extracellular vesicle–mimetic nanovesicles by extruding NP cells to create a miRNA therapeutic system integrated with miR-1249 mimics (NV-mimics). In vivo studies demonstrated that local injection of NV-mimics effectively alleviated the progression of compression-induced IVDD, preserving the water content and height of the discs, conserving NP tissues, and improving the metabolic balance of the ECM.

## 13. Others

In addition to the above common nanocarriers, various other biomaterials are used for IVD repair. Sun et al. [169] developed carbonized Mn-containing nanodots (MCDs), a class of ROS-scavenging catalytic biomaterials designed to mitigate pyroptosis in NP cells, thus significantly easing the symptoms of IVDD, which showcased superior performance in neutralizing intracellular ROS and reestablishing equilibrium within the NP microenvironment, outperforming traditional antioxidants like N-acetylcysteine. Yu et al. [170] designed an isoginkgetin-loaded, ROS-responsive delivery system based on diselenide block copolymers. This controlled-release system not only efficiently scavenged ROS in IVD but also enhanced autophagy in NP cells, thereby inhibiting ECM degradation and cell apoptosis, which showed significant therapeutic effects in a rat model of IVDD. In addition, Wang et al. [171] developed a novel approach using low-dose celecoxib-loaded polycaprolactone (PCL) nanofibers to reverse IVDD, which maintained prostaglandin E2 (PGE2) at physiological levels and promoted the expression of chondroitin sulfate synthase 3 (CHSY3), offering a new strategy for the treatment of IVDD.

## 14. Conclusions

At present, the incidence of LBP remains high, with complex etiology often related to IVDD. IVDD leads to reduced disc height and inadequate support, along with inflammation and ROS accumulation in NP. Suitable biomaterials can provide mechanical support for IVDD while guiding the regeneration of NP tissue. Additionally, these materials can carry cells, drugs, or cytokines to further promote IVD recovery. By summarizing previous preclinical studies, we discuss various biomaterials, including natural hydrogels (e.g., hyaluronic acid, chitosan, collagen/gelatin, alginate, silk fibroin), synthetic hydrogels (e.g., PEG, PVA, PAA, PLA/PLGA), microspheres, microneedles, microfluidic chips, electrospinning, electrospraying, three-dimensional bioprinting carriers, and nanocarriers (e.g., liposomes, nanomicelles, nanozymes, extracellular vesicles) in terms of their physicochemical properties and IVD repair abilities. Numerous studies have demonstrated that biocompatibility, biodegradability, biological efficacy, and the ability to maintain mechanical properties are essential for IVDD repair biomaterials. Using a single material (especially natural hydrogels) to repair IVD often lacks sufficient mechanical support to maintain the overall spinal structure, making it difficult to realize subsequent biological functions. Forming a crosslinked network with synthetic hydrogels is currently a hot topic in applications. These materials, which aim to reverse the fate of IVDs, are expected to address the limitations of current conservative and surgical treatments for IVDD, improve patients’ quality of life, and ultimately lead to fundamental changes in IVDD treatment.

## 15. Limitation

While the aforementioned therapies show promise, they are still far from clinical application. Several potential directions for IVDD biomaterials need to be explored. Firstly, current research is primarily in the cell and animal experiment stages, with the majority of studies using the SD rat coccygeal vertebra acupuncture model for IVDD. Only a few studies have used other animal models, such as New Zealand rabbits, beagles, and goats, indicating a significant gap before these findings can be translated into clinical practice. Secondly, accurately delivering biomaterials into the IVD remains a critical challenge. Injectable hydrogels are commonly used, but their injection can damage the integrity of the AF, potentially exacerbating IVDD. The injectability of hydrogels often conflicts with mechanical properties; that is, materials that provide robust support are difficult to inject into the lesion site. Simultaneously, achieving precise injection of the appropriate amount of hydrogel into the confined space of the rat tail vertebrae poses a significant challenge. Although photocurable hydrogels are widely used in tissue engineering, their properties also conflict with the requirements for injectability. Spinal channel surgery on experimental animals might offer a solution to this issue. Lastly, most IVDD patients primarily complain of LBP, making pain relief a major concern. The role of IVD repair materials in addressing discogenic pain is gaining attention, but research in this area is still limited. Challenges include establishing reliable animal models and setting appropriate evaluation criteria, both of which are crucial for future clinical applications.

## Data Availability

The data presented in this study are available in this article.
